# Effect of Crestal Position on Bone–Implant Stress Interface of Three-Implant Splinted Prostheses: A Finite Element Analysis

**DOI:** 10.3390/ma18143344

**Published:** 2025-07-16

**Authors:** Mario Ceddia, Giulia Marchioli, Tea Romasco, Luca Comuzzi, Adriano Piattelli, Douglas A. Deporter, Natalia Di Pietro, Bartolomeo Trentadue

**Affiliations:** 1 Department of Mechanics, Mathematics and Management, Polytechnic University of Bari, 70125 Bari, Italy; marioceddia1998@gmail.com (M.C.); bartolomeo.trentadue@poliba.it (B.T.); 2 Department of Medical, Oral and Biotechnological Sciences, “G. d’Annunzio” University of Chieti-Pescara, 66100 Chieti, Italy; giulia.marchioli@phd.unich.it (G.M.); tea.romasco@unich.it (T.R.); 3 Independent Researcher, 31020 San Vendemiano, Italy; luca.comuzzi@gmail.com; 4 School of Dentistry, Saint Camillus International University of Health and Medical Sciences, 00131 Rome, Italy; apiattelli51@gmail.com; 5 Facultad de Medicina, UCAM Universidad Católica San Antonio de Murcia, 30107 Murcia, Spain; 6 Department of Periodontics, Faculty of Dentistry, University of Toronto, Toronto, ON M5G 1G6, Canada; douglas.deporter@dentistry.utoronto.ca

**Keywords:** finite element analysis, dental implants, biomechanics, bone–implant interface, stress distribution, vertical misalignment, implant design, cortical bone loading

## Abstract

Optimizing stress distribution at the bone–implant interface is critical to enhancing the long-term biomechanical performance of dental implant systems. Vertical misalignment between splinted implants can result in elevated localized stresses, increasing the risk of material degradation and peri-implant bone resorption. This study employs three-dimensional finite element analysis (FEA) to evaluate the mechanical response of peri-implant bone under oblique loading, focusing on how variations in vertical implant platform alignment influence stress transmission. Four implant configurations with different vertical placements were modeled: (A) all crestal, (B) central subcrestal with lateral crestal, (C) lateral subcrestal with central crestal, and (D) all subcrestal. A 400 N oblique load was applied at 45° simulated masticatory forces. Von Mises stress distributions were analyzed in both cortical and trabecular bone, with a physiological threshold of 100 MPa considered for cortical bone. Among the models, configuration B exhibited the highest cortical stress, exceeding the physiological threshold. In contrast, configurations with uniform vertical positioning, particularly model D, demonstrated more favorable stress dispersion and lower peak values. Stress concentrations were consistently observed at the implant–abutment interface across all configurations, identifying this area as critical for design improvements. These findings underscore the importance of precise vertical alignment in implant-supported restorations to minimize stress concentrations and improve the mechanical reliability of dental implants. The results provide valuable insights for the development of next-generation implant systems with enhanced biomechanical integration and material performance under functional loading.

## 1. Introduction

Dental implants represent a reliable and widely adopted solution for tooth replacement, with over two million units placed annually in the United States and global utilization projected to reach 23% by 2026 [1,2,3,4,5]. This increasing demand is fueled by continuous advancements in implant design, surgical protocols, and biomaterials, alongside growing patient awareness of the long-term functional and esthetic benefits associated with implant-supported rehabilitation [6]. Compared to removable prostheses, dental implants offer superior outcomes in terms of masticatory efficiency, stability, and patient satisfaction. Demographic factors such as population aging, the prevalence of edentulism, and systemic health conditions further contribute to the expanding clinical need. The long-term success of implant therapy is multifactorial, influenced by bone quality, implant geometry, loading strategies, and patient-specific risk factors.

Recent scientific efforts have increasingly focused on enhancing osseointegration through surface modifications, improving material properties, and integrating digital workflows, collectively contributing to higher survival rates and a reduction in biological and mechanical complications [7,8,9]. The integration of biomechanical principles with biological considerations remains fundamental to optimizing treatment outcomes across a heterogeneous patient population.

The success and predictability of implant treatment, however, depends on many factors, including primary implant stability, the establishment of osseointegration with direct bone-to-implant contact, and the quantity and/or quality of supporting bone [6,7,8,9,10]. Moreover, occlusal forces and potential occlusal overload on implant-supported restorations can significantly affect long-term outcomes including crestal bone loss (CBL), gingival recession [11,12], and implant failure.

Crestal bone loss around dental implants and its impact have been extensively studied and discussed in the literature [13,14,15,16]. While the concept of osseointegration was originally introduced by Brånemark, the quantitative criteria for implant success—specifically, marginal bone loss of up to 2 mm during the first year in function and no more than 0.2 mm annually thereafter—were later established by Albrektsson and colleagues [17].

One of the primary etiological factors for crestal bone loss (CBL) is the mechanical stress surpassing physiological thresholds following occlusal loading [18,19]. Contributory factors to occlusal overload encompass bone quality and implant-specific parameters such as number, length, connection type, spatial distribution, and angulation. Prosthetic design characteristics—including open interproximal contacts, static and dynamic occlusal schemes, premature contacts, cantilevers, splinting, and crown-to-implant ratios—also significantly influence stress distribution, as do parafunctional behaviors like bruxism [6,13,20,21].

Beyond biomechanical considerations, biological and patient-related factors critically affect CBL risk assessment. Inadequate plaque control, peri-implant mucositis, and a prior history of periodontitis have been consistently associated with increased marginal bone loss and implant complications. Additionally, systemic conditions such as diabetes mellitus, osteoporosis, and smoking impair healing and osseointegration processes, thereby exacerbating the biomechanical impact of occlusal stresses.

A comprehensive treatment approach that integrates mechanical, biological, and behavioral considerations is therefore essential to minimize the risk of crestal bone loss and enhance implant longevity. The direction of loading significantly affects stress distribution in implant-supported prostheses. While axial forces are mainly compressive, horizontal and oblique loads introduce lateral and torsional stresses that can compromise the prosthesis or bone–implant interface if excessive [22,23,24].

Mechanical overloading is one of the main risk factors associated with implant failure in prosthetic dentistry [25,26]. A common approach to reduce this risk involves the use of splinted restorations, which are designed to distribute occlusal forces and mechanical stresses more uniformly across multiple implants [27,28,29].

Due to the difficulty of acquiring in vivo stress data, finite element analysis (FEA) has become a widely adopted tool for investigating stress and strain distributions in dental biomechanics [30,31]. Beyond quantifying mechanical loads, FEA enables the prediction of tissue response under various loading conditions, in both healthy and pathological scenarios. It is also extensively used in the development of medical devices, offering insights into their function and identifying potential complications before clinical implementation.

In implant dentistry, numerous FEA-based studies have explored the mechanical behavior of implant supported prostheses under functional loading, focusing primarily on stress and strain fields at the implant and peri-implant bone level [32]. However, mechanical stimuli also induce bone remodeling processes at the microscopic scale, which can significantly alter the stress distribution at the bone–implant interface, ultimately affecting the long-term stability of the implant system [33,34,35].

The modeling process of the FEA model typically involves the reconstruction of mandibular or maxillary bone geometry from diagnostic imaging, such as computed tomography (CT). Although CT offers detailed anatomical data, the high radiation dose remains a concern, particularly for non-critical evaluations. Nevertheless, several studies have shown that modeling bone tissue at the macroscopic level distinguishing only between cortical and trabecular regions can still yield accurate predictions of mechanical behavior [36,37].

In this study, bone geometry was simplified as a rectangular block comprising an outer cortical layer and an internal trabecular core. Mechanical properties were assigned to all relevant materials, including bone and prosthetic components. A three-dimensional finite element mesh was generated, and physiological loading and boundary conditions were applied to simulate masticatory function. The simulation results, including stress and strain distributions, were compared to available experimental and clinical data for validation.

Previous work has demonstrated strong agreement between FEA predictions and empirical measurements [38,39]. For example, Ingawale et al. [40] validated FEA models of the human mandible using data from the biomechanical testing of fresh frozen cadaver specimens, reporting high correlation coefficients (R^2^ = 0.964–0.999) for predicted vs. measured principal strains. Similarly, comparative studies using photoelastic analysis have confirmed the reliability of FEA in replicating stress distribution patterns under compressive loads [41].

Regarding splinted implants, several studies have assessed the influence of implant number and positioning on peri-implant stress transmission. Andrade et al. [42] and Shigemitsu et al. [27], for instance, reported that splinted prostheses exhibit more favorable biomechanical behavior and lower stress concentrations compared to non-splinted single-unit restorations.

Using photoelastic modeling to analyze load transfer, Guichet et al. [43] concluded that stress localizes at interproximal contacts of non-splinted implants, while loads are evenly distributed amongst implants supporting splinted prostheses, with a particularly significant load-sharing effect on the central implant of three-implant supported restorations. FEA work by Lemos et al. [44] evaluated stress distribution around splinted Morse taper implants again concluding that splinting promoted better distribution.

Similarly, Toniollo et al. [45] reported that increasing the number of splinted implants from two to three resulted in further stress reduction. Regarding prosthetic designs, if the emergence profiles of implant restorations including bridges are not properly designed to facilitate daily hygiene and professional implant maintenance, the risk of crestal bone loss leading to implant failure increases significantly [46,47]. According to Yi et al. [48], splinted implants have a higher risk of peri-implantitis compared to non-splinted ones, especially when convex crown profiles are present. Indeed, the risk of peri-implantitis was found to be 4.66 times higher with splinting. In a subsequent study, the same authors evaluated biological complications with splinting over a 15-year period involving 888 implants in 423 patients. They observed complications in 45.4% of splinted implants compared to 26% of non-splinted implants. In their clinical study, Wagenberg et al. [49] also reported statistically significant differences in crestal bone loss between splinted (−0.50 ± 0.8 mm) and non-splinted implants (−0.30 ± 0.65 mm). However, splinted prostheses supported by three implants showed less crestal bone loss than those supported by two implants.

Although three-implant splinted prostheses may reduce the risk of mechanical complications, the central implant may be particularly prone to peri-implantitis. Lin et al. [5] reported a higher incidence of peri-implant bone loss in patients with adjacent implants placed with different vertical platform levels. In splinted configurations, implants with a vertical mismatch of ≥0.5 mm had a significantly higher probability of experiencing bone resorption ≥1 mm.

One approach to reduce biological complications of crestal bone loss is to place implants subcrestally [50,51].

A finite element analysis (FEA) study conducted by Di Pietro et al. [52] demonstrated that subcrestal implant placement is biomechanically more effective in distributing stress and may help reduce peri-implant bone resorption, thereby promoting improved long-term implant stability.

This positioning not only improves stress distribution but also helps isolate the implant–abutment interface from oral pathogens, thereby reducing the risk of inflammation or infection. However, to date, no finite element analysis (FEA) studies have examined the biomechanical impact of vertical implant positioning in splinted prosthetic restorations relative to the alveolar crest. The present study addresses this gap by providing a novel analysis that offers clinically relevant insights to optimize implant placement strategies and improve long-term outcomes. The null hypothesis states that implant positioning relative to the alveolar crest does not influence the stress distribution.

## 2. Materials and Methods

**Figure 1:**
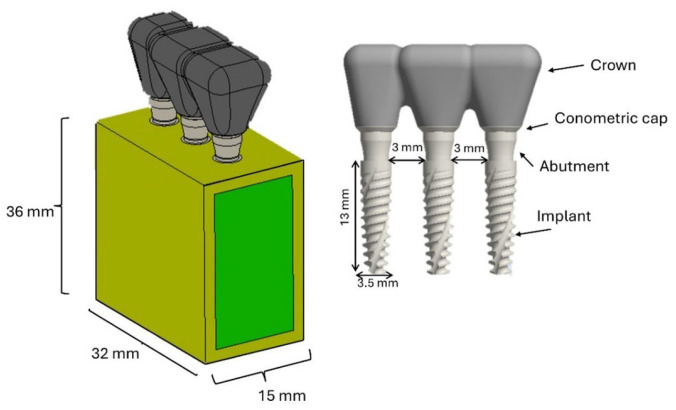
Three-dimensional representation of the components: prosthesis and bone segment.

A posterior segment of human mandible, including premolar and molar regions, was used in modeling. The segment measured 32 mm mesiodistally and 15 mm buccolingually and had a bone height of 36 mm, including a cortical thickness of 1.5 mm [53,54]. Implants were supplied by AoN Implants (AoN Implants Srl, Grisignano di Zocco, Italy) and were modeled in three dimensions using Autodesk Inventor 3D modeling software (Autodesk Inventor 2023, San Francisco, CA, USA). Each implant had an outer diameter of 3.5 mm and a length of 13 mm. Their geometry featured single, sharp, aggressive threads and a flat apical end. Three implants were splinted with an inter-implant distance of 3 mm and restored with prosthetic crowns retained by a conometric connection between coping and abutment. The crowns were cemented to the copings with a cement layer thickness of 40 μm (Figure 1).

**Figure 2:**
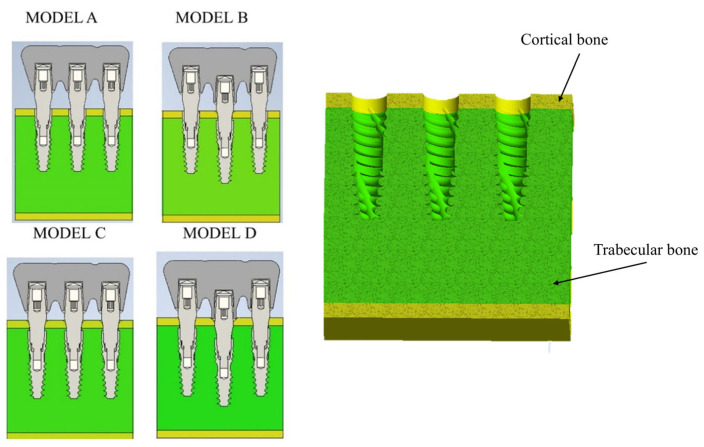
Different models analyzed based on the placement of three implants in relation to the bone crest.

The implants were placed in the bone block according to the configurations shown in Figure 2:

Model A: Three splinted implants positioned crestally.

Model B: Three splinted implants with the mesial and distal implants placed crestally, and the central implant positioned 2 mm subcrestally.

Model C: Three splinted implants all positioned 2 mm subcrestally.

Model D: Three splinted implants with the mesial and distal implants placed 2 mm subcrestally, and the central implant positioned 3 mm from the crestal cortical surface.

The cortical and trabecular bone was modeled as an anisotropic material. This choice is justified by the findings of a study conducted by Taheri et al. [55], which demonstrated that using isotropic models for bone may underestimate stress values or fail to capture mechanical phenomena critical to implant stability. Furthermore, Gasik et al. [37] developed numerical models incorporating heterogeneous bone density obtained from CT imaging, showing that anisotropic models dependent on bone density yield higher and more realistic stress distributions compared to homogeneous ones. Therefore, modeling bone as an anisotropic material allows for a more accurate representation of its actual mechanical response under load. All elastic constants used for both cortical and trabecular bone are listed in Table 1 to ensure proper material definition. In contrast, the implant, crown, and cement materials were assumed to be isotropic and linearly elastic [30,56,57,58,59,60].

### 2.1. FEA Modeling

**Figure 3:**
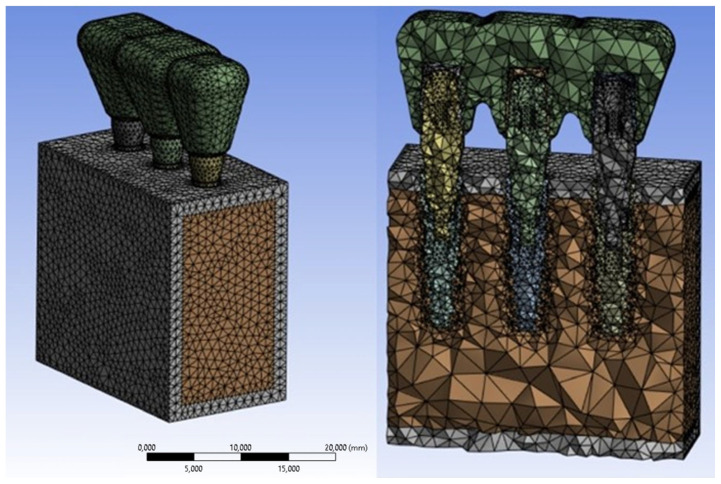
FEA modeling of the bone block and splinted implants.

Using the FEA software ANSYS Workbench R2023, the 3D model of the bone block and implants was discretized with tetrahedral elements. This choice was made due to the ability of tetrahedral elements to more accurately represent stress and deformation in complex geometries. A mesh sensitivity analysis was then performed to determine the optimal element size. Three mesh densities (1.0 mm, 0.75 mm, and 0.5 mm) were tested under identical boundary conditions and loading scenarios. The results showed that refining the mesh from 1.0 mm to 0.75 mm led to significant changes in stress values, especially around the implant neck. However, further refinement to 0.5 mm resulted in minimal differences (<5%), indicating that mesh convergence had been achieved. The 0.5 mm mesh was therefore selected as the optimal configuration, providing a good balance between computational efficiency and numerical accuracy. The final mesh consisted of 30,623 elements for the implants, 14,568 for the cortical bone, and 20,379 for the trabecular bone (Figure 3). 

**Figure 4:**
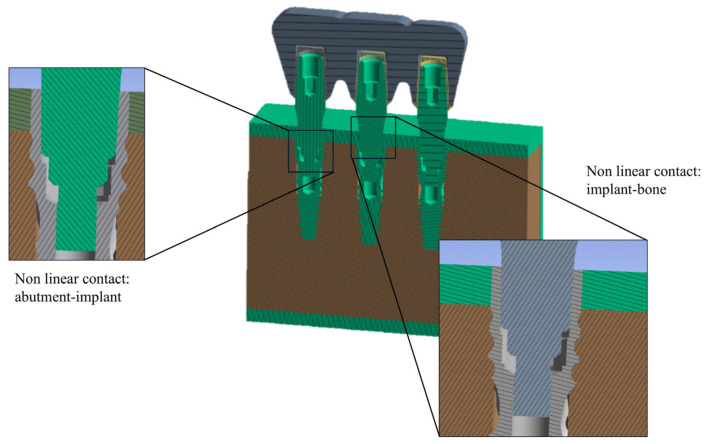
Non-linear contact interfaces.

Two critical non-linear contact interfaces were defined: the implant–bone and the implant–abutment interfaces. The following friction coefficients were applied: 0.3 for all titanium–titanium contacts, 0.65 for the cortical bone–implant interface, and 0.77 for the trabecular bone–implant interface (Figure 4). Complete osseointegration of the implants was assumed [61,62,63,64].

### 2.2. Load and Boundary Conditions

**Figure 5:**
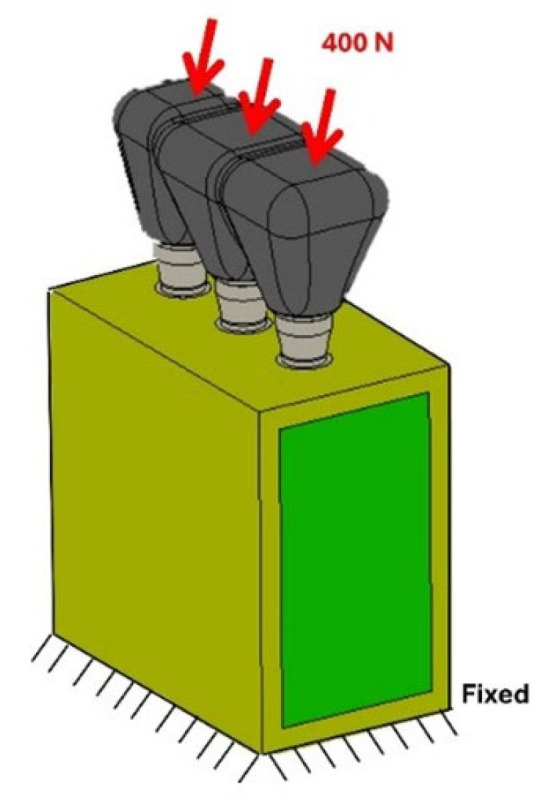
Loading and boundary conditions.

Loading conditions involved the application of a 400 N oblique load at a 45° angle on the occlusal surface of the crowns [65]. The force was directed buccally and distally. Subsequently, the bone block was constrained in all directions (Figure 5).

Von Mises stress was used to evaluate stress distributions within each implant/prosthesis/bone system. Stress values were compared against a physiological yield limit of 100 MPa for mandibular bone [66]. The average computation time per model was 26 min, performed on a 4-CPU system (2× Intel^®^ Core i7 13th generation, 14 cores each) with 16 GB of RAM. A static structural analysis was carried out to simulate the mechanical behavior of the implant-supported restoration under masticatory load. This approach assumes that the applied forces are constant or change slowly over time and neglects any inertial or damping effects that would be relevant in a dynamic context. The static analysis was deemed appropriate for simulating normal occlusal loading during habitual mastication, which typically occurs within quasi-static conditions. This method enables an accurate assessment of how the applied forces are transmitted through the prosthetic components and into the surrounding bone. The resulting stress distribution helps identify potential risk areas for mechanical failure or excessive bone remodeling. Static analysis is widely used in biomechanical studies to predict implant performance under functional loads, providing valuable insights into both the short-term mechanical response and the long-term clinical implications of prosthetic design and implant positioning.

## 3. Results

**Figure 6:**
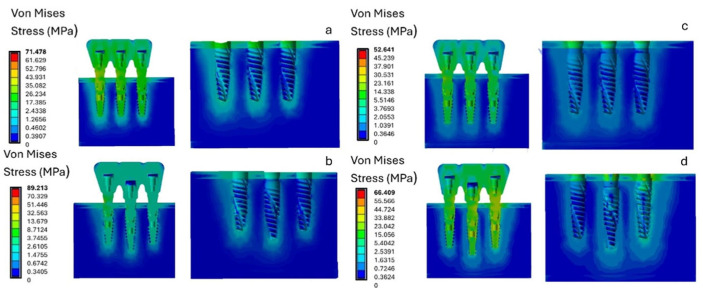
Von Mises stress analysis of the four models (**a**–**d**) under a vertical load of 400 N.

This study demonstrated that stress concentration was influenced by the misalignment of the prosthetic platform positions of the three-implant splinted restorations. Specifically, under vertical loading conditions, model B, with the central implant positioned below the crestal margin and the two lateral implants (mesial and distal) at crestal level, showed the highest stress value (89.213 MPa). This value exceeded those observed in model A (71.478 MPa), model D (66.409 MPa), and model C (52.641 MPa), as shown in Figure 6. The maximum stress values were recorded in the connection area between the abutment and the implant.

**Figure 7:**
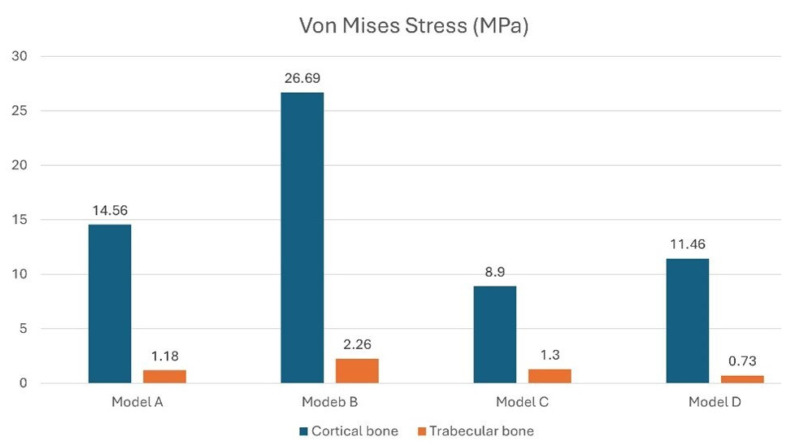
Von Mises stress analysis in cortical and trabecular bone under a vertical load of 400 N.

With a vertical load of 400 N, stress analyses on the cortical bone revealed that the crestal regions experienced the highest stress levels. Specifically, as shown in Figure 7, model B had a stress on the cortical bone of approximately 26.69 MPa at the central implant, while configuration A had a stress of 14.56 MPa uniform across all three implants. This initial analysis indicated that both the crestal placement of three adjacent splinted implants (Model A) and the misaligned placement of the prosthetic platform (Model B) resulted in higher stress levels. In contrast, configurations with all implants placed subcrestally (Models C and D) showed lower stress values. The maximum stress on the trabecular bone was recorded at the apex of the implant. For model A, the maximum value was 1.18 MPa for the mesial implant. Model B recorded a stress value of 2.26 MPa on the trabecular bone for the central implant. Models C and D recorded lower stress values: 1.3 MPa for the central implant in model C and 0.73 MPa in the same area in model D.

### 3.1. Effect of Inclined Load

**Figure 8:**
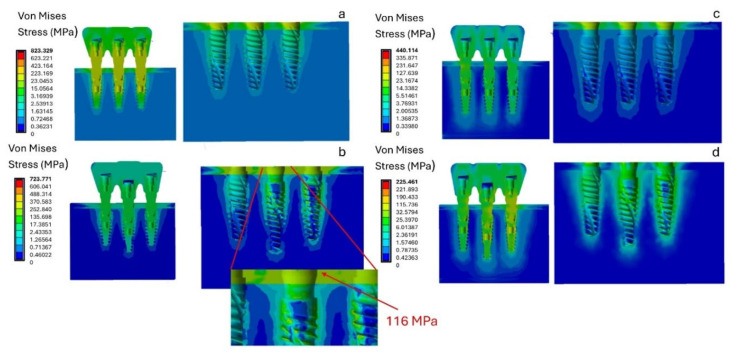
Von Mises stress analysis of the four models (**a**–**d**) under a 400 N inclined load.

Oblique loading of the peri-implant bone produced higher local stresses distributed over a larger area compared to vertical loading, with stress peaks concentrated primarily near the crestal region of the implant. Furthermore, while vertical loading produced a more homogeneous stress distribution at the trabecular bone–implant interface, oblique loading caused most of the stress to be concentrated at the top of the implant, as shown in Figure 8. This altered the typical stress distribution within the bone. The biomechanical explanation lies mainly in the increased bending and torsional forces induced by oblique loading. This effect is amplified when the prosthetic platform positions of the three implants are not aligned or when all implants are positioned at the crestal level. In fact, as shown in Figure 8, the highest stress values were recorded for model A (823.329 MPa) and model B (723.721 MPa).

**Figure 9:**
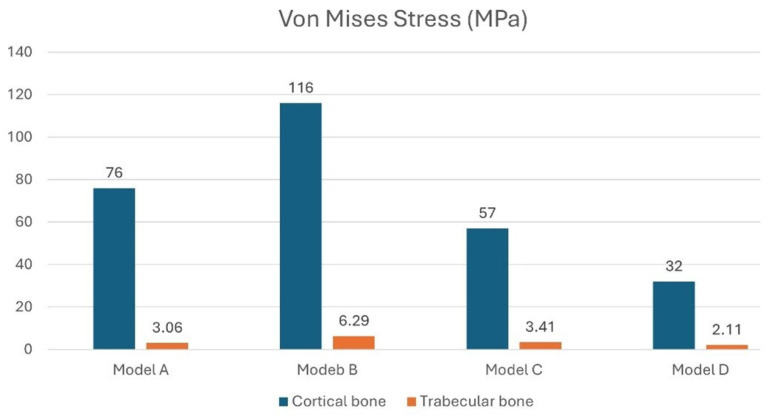
Von Mises stress analysis in cortical and trabecular bone under a 400 N inclined load.

Figure 8 shows that model B, with the central implant positioned subcrestally relative to the two lateral implants positioned crestally, has a cortical bone stress value of about 116 MPa at the central implant. This stress value is close to the 120 MPa physiological stress limit of cortical bone [67]. This suggests that this configuration may exceed the physiological threshold of bone, indicating a higher risk of overload and potential bone damage. Furthermore, the increased stress under oblique loading promotes localized activation of bone remodeling, which is more pronounced in cortical bone due to its higher vascularization, thereby triggering the resorption process. Figure 9 shows that stress on both cortical and trabecular bone can be minimized by placing all three implants subcrestally, with the central implant positioned 2 mm below the alveolar crest (model D). This configuration reduces stress on the cortical bone to 32 MPa and minimizes stress on the trabecular bone to 2.11 MPa.

Therefore, analyzing the stress on the bone, model B exhibits higher stress at the bone–implant interface under both vertical and oblique loading conditions. In contrast, the subcrestal positioning of all three implants, represented by models C and D, shows lower stress values that remain within the physiological limits for both cortical and trabecular bone.

### 3.2. Stress on the Implants

Regarding implant stress, the highest values occur in the case of oblique loading due to the additional bending stress generated on the abutment neck.

**Figure 10:**
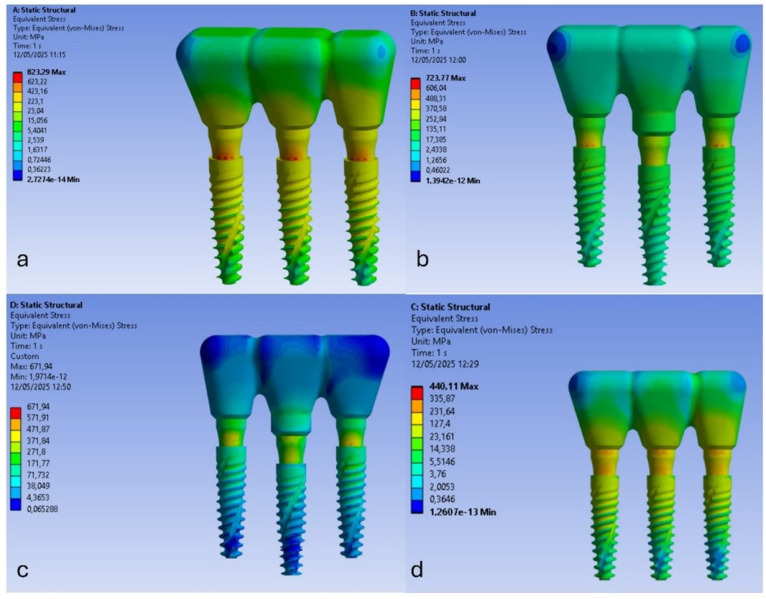
Von Mises stress on the implants under a 400 N oblique load for different models (**a**–**d**).

Specifically, the maximum stress was observed when the implants were positioned at the crestal level (Model A), with a peak of 676 MPa, and when the central implant was positioned subcrestal (Model B), reaching 702 MPa at the connection area between the abutment and the implant, as shown in Figure 10.

## 4. Discussion

Preserving the integrity of the peri-implant marginal bone remains a critical clinical challenge. Multiple biomechanical factors influence bone behavior, often initiating bone resorption processes that can compromise implant stability and long-term success [68,69]. For instance, it is unclear whether loads applied to prostheses supported by multiple implants with different implant connection types affect bone resorption [70,71]. A study by Tonin et al. [72] found that using conometric connections with prosthetic structures fabricated by laser welding or CAD/CAM resulted in lower stress levels at the bone crest, suggesting greater marginal bone tissue preservation.

In contrast, groups using tungsten inert gas welding showed high stress levels, particularly in the crest region, for both hexagonal external and conometric connections. This indicates a potential increase in the risk of bone resorption. Furthermore, the timing between implant placement and the onset of functional loading plays a pivotal role in the preservation of peri-implant bone tissue. For example, Krawiec et al. [73] demonstrated that immediate loading led to reduced crestal bone resorption compared to loading delayed by 12 months. Nevertheless, biomechanical stress remains a primary factor contributing to early implant failure and marginal bone loss [13]. The use of multiple splinted implants is well supported in the literature, as it results in lower stress transmission to peri-implant tissues compared to single implants [74,75,76,77]. A finite element analysis (FEA) study [77] investigated the influence of implant number on peri-implant biomechanical behavior. Findings revealed that while two implants generated increased stress on both peri-implant bone and the abutment, the addition of a third implant significantly mitigated these stresses. Specifically, the incorporation of a central implant within a three-implant-supported prosthesis reduced stress concentration around the implant, thereby fostering bone tissue preservation and minimizing the risk of adverse remodeling and prosthetic component failure.

Another study found that two 4.1 mm diameter implants are sufficient to support partial prostheses without compromising the integrity of the cortical bone. Nevertheless, the addition of a central implant further decreases stress levels [78].

To date, no numerical investigations have evaluated the combined influence of vertical discrepancy and the position of splinted implants relative to the alveolar crest on stress distribution in peri-implant tissues. Existing research on vertical placement has focused exclusively on single implants [51,79,80,81]. For example, Li et al. [82] used FEA to show that placing implants 0.5 mm below the alveolar crest results in less bone deformation compared to positioning them 1 mm above it. From a clinical perspective, several studies have linked vertical discrepancies between splinted implants to complications such as marginal bone resorption and peri-implantitis. In a 15-year retrospective study, Yi et al. [83] reported that the central implant among three contiguous connected implants exhibited a significantly higher risk of marginal bone loss and peri-implantitis compared to mesial, distal, or single implants. Similarly, Prete et al. [84] confirmed increased bone loss around the central implant positioned at the crestal level, whereas the two lateral implants were placed subcrestally. Lin et al. [5] conducted a comparative analysis demonstrating that splinted prostheses with vertical discrepancies of at least 0.5 mm are associated with greater bone loss than non-connected implants. Notably, in over 90% of cases, the most apical implant exhibited the least peri-implant bone loss. These findings suggest that such complications are predominantly driven by inflammatory processes, including infection and periodontitis.

In our study, we examined the null hypothesis that the vertical positioning of splinted implants relative to the alveolar ridge alters the normal distribution of stresses using finite element analysis (FEA). We simulated four configurations with different misalignments of the prosthetic platforms.

Our results reject the null hypothesis because the models with completely subcrestal implants (models C and D) showed reduced stress concentrations on the implants and peri-implant bone. Conversely, model B, which had the mesial and distal implants at the crestal level and the central implant subcrestal, generated stress values on the cortical bone of up to 116 MPa. This could potentially exceed the bone’s physiological tolerance threshold. Therefore, this could trigger bone remodeling and resorption due to overload.

### Limitations

This study is subject to certain limitations, primarily concerning the potential overgeneralization of the mechanical properties attributed to the analyzed bone tissues. In particular, cortical and trabecular bone were represented as homogeneous, anisotropic materials. However, it is well established in the literature that bone exhibits intrinsic heterogeneity due to its complex internal microarchitecture [85,86,87]. This modeling simplification may limit the extent to which the present findings can be generalized to in vivo conditions

Recently, finite element (FE) models have been developed from micro-CT images that represent the specific architecture of the sample, including both cortical and trabecular bone. The microCT images were converted into a voxel-based FE model in which each element was assigned the same elastic modulus [88]. Although microCT allows for high accuracy in both internal and external geometry, it has limitations related to high computational cost. This is because modeling bone architecture in detail requires many elements (e.g., 2.1 million quadratic tetrahedral elements with typical dimensions of 0.1 mm). Moreover, modeling a single sample using microCT may not capture interindividual or anatomical variability. Nonetheless, simplified models offer the advantage of easier parameterization and broader applicability [89].

It is important to note that the loading conditions in this study were assumed to be static, whereas implants are subjected to variable and cyclic dynamic forces in clinical scenarios [90].

Despite these limitations, numerous investigations have reported a strong concordance between finite element analysis (FEA) outcomes and both in vivo and in vitro experimental data. For instance, Ceddia et al. [91] conducted a comparative study integrating FEA with experimental testing to evaluate primary stability, demonstrating a high level of agreement between the two approaches, with an average prediction error of only 1.27% for the FEA model. Nonetheless, further clinical and laboratory studies employing extensometric techniques are warranted to corroborate and validate the findings of the present study.

## 5. Conclusions

This finite element analysis revealed that the vertical positioning of three adjacent implants within a splinted prosthetic framework has a substantial impact on stress distribution at the bone–implant interface. Uniform implant placement, whether at the crestal or subcrestal level, was associated with lower and more evenly distributed peri-implant stresses, remaining within physiological limits and thereby minimizing the risk of marginal bone resorption. Conversely, vertical discrepancies—particularly configurations in which the central implant is placed subcrestally while the lateral implants are crestally positioned—resulted in localized peak stresses in the cortical bone that exceeded physiological thresholds, potentially eliciting unfavorable bone remodeling and increasing the risk of biological complications.

These findings highlight the importance of consistent vertical alignment and appropriate splinting strategies during prosthetic planning.

Subcrestal implant placement appears to be a biomechanically favorable option, contributing to reduced peri-implant stress and enhanced stability. Overall, adopting a uniform vertical implant configuration combined with splinted restorations may mitigate mechanical overload and support long-term clinical success. These biomechanical insights offer a useful framework for guiding future implant design and clinical protocols aimed at optimizing functional and biological outcomes.

## Figures and Tables

**Table 1:** Mechanical characteristics of the materials.

Material	Young’s Modulus E (MPa)	Poisson’s Ratio ν	Shear Modulus G (MPa)
Cortical bone	Ex = 12,600 Ey = 12,600 Ez = 19,400	$\nu_{xy}=0.3$ $\nu_{yz}=0.253$ $\nu_{xz}=0.253$ $\nu_{yz}=0.3$ $\nu_{zy}=0.39$ $\nu_{zx}=0.39$	Gxy = 4850 Gyz = 5700 Gxz = 5700
Cancellous bone	Ex = 1148 Ey = 210 Ez = 1148	$\nu_{xy}=0.055$ $\nu_{yz}=0.01$ $\nu_{xz}=0.322$ $\nu_{yz}=0.01$ $\nu_{zy}=0.055$ $\nu_{zx}=0.322$	Gxy = 68 Gyz = 68 Gxz = 434
Titanium (Ti6Al4V)	110,000	0.35	
Ceramic (ZrO_2_)	150,000	0.3	
Cement	280	0.3	

The vectors of x, y, and z indicate the buccolingual, infero-superior, and mesiodistal direction, respectively.

## Data Availability

The original contributions presented in this study are included in the article. Further inquiries can be directed to the corresponding author.
